# Supplementary material for: Targeting Cardiac Metabolism in Heart Failure with PPARα Agonists: A Review of Preclinical and Clinical Evidence
Source: Biomedicines. 2025 Aug 26;13(9):2080. doi: 10.3390/biomedicines13092080 (PMC12467676; doi:10.3390/biomedicines13092080)
Supplement: Supplementary file 1 [file biomedicines-13-02080-s001.zip › biomedicines-3759372-supplementary.pdf]

Supplemental material

The search strategy of preclinical studies

Ovid Medline search

1. "heart failure".tw,kf.
2. exp heart failure/
3. "diabet*".tw,kf.
4. exp diabetes mellitus/
5. ("fenofibrate" or "gemfibrozil" or "clofibrate" or "ciprofibrate" or "bezafibrate" or "pemafibrate" or "PPARalpha" or "PPARα" or "PPAR-alpha" or "PPAR-α").tw,kf.
6. Peroxisome proliferator activated receptor alpha/
7. exp animals/
8. ("rat" or "rats" or "mouse" or "mice" or "swine" or "porcine" or "murine" or "sheep" or "lambs" or "pigs" or "piglets" or "rabbit" or "rabbits" or "cat" or "cats" or "dog" or "dogs" or "cattle" or "bovine" or "monkey" or "monkeys" or "trout" or "marmoset").tw,kf.
9. ("pre-clinical" or "preclinical" or "in vivo" or "in vitro").tw,kf.
10. 1 or 2
11. 3 or 4
12. 5 or 6
13. 7 or 8 or 9
14. 10 and 11 and 12 and 13

EMBASE search

1. "heart failure".tw,kf.
2. exp heart failure/
3. "diabet*".tw,kf.
4. exp diabetes mellitus/
5. ("fenofibrate" or "gemfibrozil" or "clofibrate" or "ciprofibrate" or "bezafibrate" or "pemafibrate" or "PPARalpha" or "PPARα" or "PPAR-alpha" or "PPAR-α").tw,kf.
6. Peroxisome proliferator activated receptor alpha/
7. ("rat" or "rats" or "mouse" or "mice" or "swine" or "porcine" or "murine" or "sheep" or "lambs" or "pigs" or "piglets" or "rabbit" or "rabbits" or "cat" or "cats" or "dog" or "dogs" or "cattle" or "bovine" or "monkey" or "monkeys" or "trout" or "marmoset").tw,kf.
8. animal experiment/
9. ("pre-clinical" or "preclinical" or "in vivo" or "in vitro").tw,kf.
10. 1 or 2
11. 3 or 4
12. 5 or 6
13. 7 or 8 or 9
14. 10 and 11 and 12 and 13

Systematic search of randomised controlled trials

The search strategy MEDLINE (OVID)

1 exp randomized controlled trial/
2 controlled clinical trial.pt.

3 randomized.ab.
4 placebo.ab.
5 clinical trials as topic/
6 randomly.ab.
7 trial.ti.
8 or/1-7
9 exp animals/ not humans/
10 8 not 9
11 heart failure.mp. or Heart Failure/
12 fibrate.mp.
13 11 and 12

The search strategy for EBASE (OVID)

1 exp randomized controlled trial/
2 Controlled clinical trial/
3 random$.ti,ab.
4 randomization/
5 intermethod comparison/
6 placebo.ti,ab.
7 (compare or compared or comparison).ti.
8 ((evaluated or evaluate or evaluating or assessed or assess) and (compare or compared or comparing or comparison)).ab.
9 (open adj label).ti,ab.
10 ((double or single or doubly or singly) adj (blind or blinded or blindly)).ti,ab.
11 double blind procedure/
12 parallel group$1.ti,ab.
13 (crossover or cross over).ti,ab.
14 ((assign$ or match or matched or allocation) adj5 (alternate or group$1 or intervention$1 or patient$1 or subject$1 or participant$1)).ti,ab.
15 (assigned or allocated).ti,ab.
16 (controlled adj7 (study or design or trial)).ti,ab.
17 (volunteer or volunteers).ti,ab.
18 human experiment/
19 trial.ti.
20 or/1-19
21 (random$ adj sampl$ adj7 ("cross section$" or questionnaire$1 or survey$ or database$1)).ti,ab. not (comparative study/ or controlled study/ or randomi?ed controlled.ti,ab. or randomly assigned.ti,ab.)
22 Cross-sectional study/ not (exp randomized controlled trial/ or controlled clinical study/ or controlled study/ or randomi?ed controlled.ti,ab. or control group$1.ti,ab.)
23 (((case adj control$) and random$) not randomi?ed controlled).ti,ab.
24 Systematic review.ti,ab. not (trial or study).ti.
25 (nonrandom$ not random$).ti,ab.
26 "random field$".ti,ab.
27 (random cluster adj3 sampl$).ti,ab.
28 (review.ab. and review.pt.) not trial.ti.
29 "we searched".ab. and (review.ti. or review.pt.)
30 "update review".ab.
31 (databases adj4 searched).ab.

32 (rat or rats or mouse or mice or swine or porcine or murine or sheep or lambs or pigs or piglets or rabbit or rabbits or cat or cats or dog or dogs or cattle or bovine or monkey or monkeys or trout or marmoset$1).ti. and animal experiment/
33 Animal experiment/ not (human experiment/ or human/)
34 or/21-33
35 20 not 34
36 35 and fibrate.mp. [mp=title, abstract, heading word, drug trade name, original title, device manufacturer, drug manufacturer, device trade name, keyword heading word, floating subheading word, candidate term word]
37 36 and heart failure.mp. [mp=title, abstract, heading word, drug trade name, original title, device manufacturer, drug manufacturer, device trade name, keyword heading word, floating subheading word, candidate term word]

The search strategy for Clincialtrials.gov

Condition or disease: Heart failure
Other terms: fibrate or fenofibrate or gemfibrozil or clofibrate

SM Table S1. Pre-clinical studies of the effect of PPARα agonists on heart failure models: experimental techniques and key limitations.

Author	Experimental techniques	Key limitations
**Young et al, 2001 [18]**	RNA extraction, qRT-PCR	No correlation of altered UCP gene expression to protein expression or activity. Limited exploration of the direct PPARα-UCP3 interaction and of functional consequences of increased UCP3 expression. No in vivo studies. Lack of long-term outcome data.
**Young et al, 2001 [19]**	Langendorff mode, RNA Extraction, qRT-PCR	No underlying mechanism explored. Lack of long-term outcome data.
**Aasum et al, 2002 [20]**	Blood serum analysis, isolated heart perfusions, measurement of ventricular function, cardiac metabolism measurements, RNA analysis, tissue TG content	No underlying molecular mechanism explored. Lack of long-term outcome data.
**Ichihara et al, 2006 [21]**	Histopathology, imaging, gene expression, transcription factor expression	Limited understanding of mechanisms. Lack of long-term outcome data.
**Morgan et al, 2006 [22]**	Echocardiography, hemodynamic measurements, metabolic products and enzyme activity, RNA extraction, qRT-PCR, Western immunoblot analysis	Did not measure myocardial fatty acid uptake or oxidation directly. Use of high fat diet as control.
**Pruimboom Brees et al, 2006 [23]**	Serum chemistry, metabolites analysis, histopathology and immunochemistry, determination of peroxisomal-oxidation and fatty acyl-CoA oxidase mRNA levels, oxidative stress	Use of overexpression model. Further studies required to elucidate whether same metabolic changes happen in healthy hearts treated with PPARα agonists. Lack of long-term outcome data.
**King et al, 2007 [24]**	Mitochondria oxygen consumption using Clark-type oxygen electrode, measurement of mitochondrial palmitate export using thin-layer chromatography, RNA extraction, quantitative RT-PCR, Western blot analysis, measurement of MTE-I and citrate synthase activities, mRNA and protein expression	No physiological measurements. Limited exploration of underlying mechanisms. Lack of long-term outcome data.
**Anne D. Hafstad et al, 2009 [25]**	Serum plasma chemistry, RT-PCR, perfused heart functional measurements	Metabolic changes in model don't completely match to diabetic changes. Limited mechanistic insight. Lack of long-term outcome data.
**Chen et al, 2010 [26]**	Histopathology, blood glucose and plasma free fatty acid, immunohistochemistry, mRNA expression in heart tissue (TNFa, adiponectin and UCP3)	Viral infection model has limited translation to human heart failure. Lack of long-term outcome data.
**Haemmerle et al, 2011 [27]**	qRT-PCR, oxygen consumption, mitochondrial investigations including respirometry, membrane potential, particle size and non-esterified thiol concentrations, western blot analysis, fatty acid oxidation, electron microscopy, PPARα measurements, tissue triglyceride analysis, measurement of cardiac glycogen, plasma analysis	Did not look at molecular nature of mediators regulating PPARα lipolysis. Lack of long-term outcome data.
**Jia et al, 2014 [28]**	Echocardiography, RNA Extraction and Real-Time PCR, Western Blot Analysis, ELISA Analysis	No underlying mechanism for how increased HMGB1 causes cardiac hypertrophy. Lack of long-term outcome data.
**Ibarra-Lara et al, 2016 [29]**	Biochemical serum measurement, western blotting, electron microscopy	No physiological measurements. Lack of long-term outcome data.
**Kaimoto et al, 2017 [12]**	Transthoracic echocardiography, RNA analysis with RT-PCR, Western blot analysis, myocardial high-energy phosphate measurement, Langendorff model,	Did not use PPARα antagonist to show that PPARα is sufficient for improvement. Measured ATP and phosphocreatine in whole heart so unclear what the exact concentrations were. Lack of long-term outcome data.
**Ibarra-Lara et al, 2019 [30]**	Echocardiogram, blood protein analysis, electron microscopy, ex vivo functional analysis including western blot, cytokine quantification	Majority of experiments ex vivo. Lack of long-term outcome data.

**Sanchez-Aguilar et al, 2023 [31]**	Serum biochemistry, western blot, histology, staining, immunodetection, immunoassay	No physiological measurements. Lack of long-term outcome data.
**Finck et al, 2002 [32]**	microPET, echocardiography, histological analysis, RNA and protein blot analysis, serum chemistries	Use of overexpression model. Mechanism involved in the increased myocardial fatty acid uptake remains unknown. Lack of long-term outcome data.
**Park et al, 2005 [33]**	Glucose and palmitate uptake, insulin signalling and function investigations, biochemical assays, protein and lipid expression, echocardiography	Use of overexpression model. Role of STAT3 not fully elicited. Lack of long-term outcome data.
**Marionneau et al, 2008 [34]**	Electrophysiological recordings, electrocardiographic recordings, RT-PCR, western blot analyses	No underlying mechanism or exploration of how altered ion channel function affects cardiac physiology. Use of overexpression model. Lack of long-term outcome data.
**Duerr et al, 2014 [35]**	Echocardiography, histology, immunohistochemistry, mRNA isolation and RT-qPCR, Western blot analysis	Use of overexpression model. Lack of long-term outcome data.
